# Postprocedural infection rate after minor surgical procedures performed with and without sterile gloves: a systematic review and meta-analysis

**DOI:** 10.1097/JS9.0000000000001993

**Published:** 2024-07-24

**Authors:** Oshan Shrestha, Sunil Basukala, Nabaraj Bhugai, Sujan Bohara, Ayush Bhatt, Niranjan Thapa, Sushanta Paudel, Niraj Joshi, Bipin Mehta, Astutee Acharya, Nirakar Shrestha

**Affiliations:** a Department of Cardiothoracic and Vascular Anaesthesiology, Manmohan Cardiothoracic Vascular and Transplant Center, Kathmandu; b Department of Cardiovascular Surgery, Shahid Gangalal National Heart Centre; c Department of Surgery, Vayodha Hospitals, Kathmandu, Nepal

**Keywords:** clean, gloves, nonsterile, procedure, sterile

## Abstract

**Introduction:**

Postprocedural infection has been a top priority for the perioperative team. The use of sterile gloves to counter this became popular and was routinely used, but randomized studies have shown that the benefit that was thought to be added by the use of sterile gloves is insignificant and that not all procedures require the use of sterile gloves.

**Methods:**

Prospective protocol registration was and electronic databases were searched without using any search filters. Screening was performed by independent reviewers, and data was extracted from selected studies. Heterogeneity was assessed by the *I* ^2^ test, and the effect model was chosen accordingly. The odds ratio was used as an effect measure as the variables in this study were dichotomous. Forest plots and funnel plots were used to give visual feedback.

**Results:**

This meta-analysis included 14 comparative studies that involved a total of 12,625 patients. Analysis of postprocedural infection outcome showed no significant difference between the procedure performed using sterile gloves and without using sterile gloves (OR: 0.88; 95% CI: 0.71–1.10; *n*=12,625; *I* ^2^=0%; *P*-value=0.26). Sensitivity analysis and subgroup analysis for randomized studies only, surgical site infection, and patients that did not receive prophylactic antibiotics showed no variations. The use of sterile gloves did not show any extra benefit for controlling infection during wound repair, excision and suturing, cystoscopy, and urinary catheterization.

**Conclusion:**

The use of sterile gloves does not have any extra benefit for preventing infections when minor surgical procedures are performed.

## Introduction

Highlights

- Postprocedural infection has been a top priority for the perioperative team.
- Clean gloves are noninferior to sterile gloves in infection control during minor procedures.
- Not all surgical procedures require sterile gloves.
- Evidence-based, diligent, and sustainable use of resources is promoted.
- These findings are limited to minor surgical procedures only.

Surgeries are supposed to have started in the Neolithic age, in a crude sense, with the gradual introduction of scientific measures in the 18th century. Surgical techniques were quite primitive and even barbaric in ancient times, with very high infection rates compared to today’s standards^1,2^. Infections are the most common adverse event after a hospital stay with about 5–10% of the incidence in developed countries. Surgical site infection (SSI) or postprocedural infections have always been a top priority of the perioperative team. Postprocedural infection refers to an infection that occurs due to the spread of microorganisms spreading to the patient’s wound after an operation or surgical procedure^3,4^. Over the course of time, the use of sterile gloves to counter the postprocedural infection rate became popular and was routinely used, while the cost of care also increased. This becomes very important for low-income countries and middle-income countries (LMICs) where resources are limited and not readily available. Studies were conducted to compare the outcomes of some surgical procedures that were performed using sterile gloves and without sterile gloves (clean gloves), and the benefit that was thought to be added by the use of sterile gloves was shown to be insignificant and showed that not all procedures require sterile gloves^5,6^. Moreover, to address the issue of rampant use of resources and reduce the effects of medical waste on the environment, the Royal College of Surgeons of the United Kingdom has developed the Intercollegiate Green Theater Checklist, which promotes diligent and sustainable use of resources^7,8^.

This systematic review and meta-analysis aim to compare the outcomes of minor surgical procedures performed with the use of sterile gloves to those performed without using sterile gloves in terms of postprocedural infection.

## Materials and methods

This systematic review and meta-analysis has followed the PRISMA guidelines (Supplemental Digital Content 1, http://links.lww.com/JS9/D174, Supplemental Digital Content 2, http://links.lww.com/JS9/D175)^9^ and the AMSTAR (Supplemental Digital Content 3, http://links.lww.com/JS9/D176) guidelines^10^.

### Protocol registration

The protocol followed in this study was prospectively registered in the International Prospective Register of Systematic Reviews (PROSPERO) and in the Registry of Systematic Reviews/Meta-Analyses.

### Search strategy

In this systematic review and meta-analysis, four electronic databases (PubMed, PubMed Central, Embase, and Scopus) were searched. Search terms like (sterile), (clean), (unsterile), (nonsterile), (gloves), (dressing), (procedure), (outpatient), and (surgery) were used with appropriate Boolean operators during the search. No search filters were used, and there were no search restrictions. The details of the search strategy and the results are available in Supplementary File 1 (Supplemental Digital Content 4, http://links.lww.com/JS9/D177).

### Inclusion criteria and exclusion criteria

All the comparative studies (randomized clinical trial, nonrandomized clinical trial, and cohort studies) that compared the outcomes of performing minor surgical procedures by using sterile gloves and without using sterile gloves were included in this study. Noncomparative studies, case reports, case series, viewpoints, and editorials were excluded in this study.

### Study selection

The results obtained from the databases search were imported to Covidence^11^ for screening purpose. In the whole screening process, three independent reviewers were involved; two were assigned to screen the studies, and any conflict that arose in the process was resolved by the third reviewer. The roles of the screening reviewers were exchanged during the title-and-abstract screening and the full-text screening phases.

### Data curation

The studies that were deemed eligible to be included in this systematic review and meta-analysis were moved to the data extraction stage. A template prepared in Word with headings of study details, population, intervention, and comparator was used for the data collection purpose. The author’s list, year of publication, demographic details, baseline characteristics, the intervention of the study, the comparator of the study, and the outcomes such as postprocedural infection, grades of infection, cost of procedure, and need for hospitalization were extracted.

### Data synthesis

Variables of categorical nature were planned to be studied with the odds ratio, and variables of continuous nature were planned to be studied with the mean difference/standardized mean difference. The heterogeneity was assessed by the *I* ^2^ test, and the effect model was chosen accordingly^12^. The fixed effect model was used for heterogeneity up to 40%, and beyond this, the random effect model was used. The results were expressed with a 95% CI, and the Forest plots were used to give visual feedback. The subgroup analysis will be performed by including randomized studies only, for surgical site infection outcome, for urological procedures, and for patients that did not receive prophylactic antibiotics.

### Risk of bias assessment

The risk of bias (ROB) tool was used for randomized studies, and the Risk of Bias in Non-randomised Studies - of Interventions (ROBINS-I) was used for nonrandomized studies to assess the risk of bias. Three reviewers were involved in the critical appraisal of the studies. The assessment of bias is shown in the Figure A and B of the Supplementary File 2 (Supplemental Digital Content 5, http://links.lww.com/JS9/D178).

### Sensitivity analysis and publication bias

The sensitivity analysis was carried out by excluding each study at a time for every outcome to see if it made any significant changes in the results. The publication bias was assessed with the help of funnel plots for the meta-analysis, which had at least 10 studies^12^.

## Results

**Figure 1:**
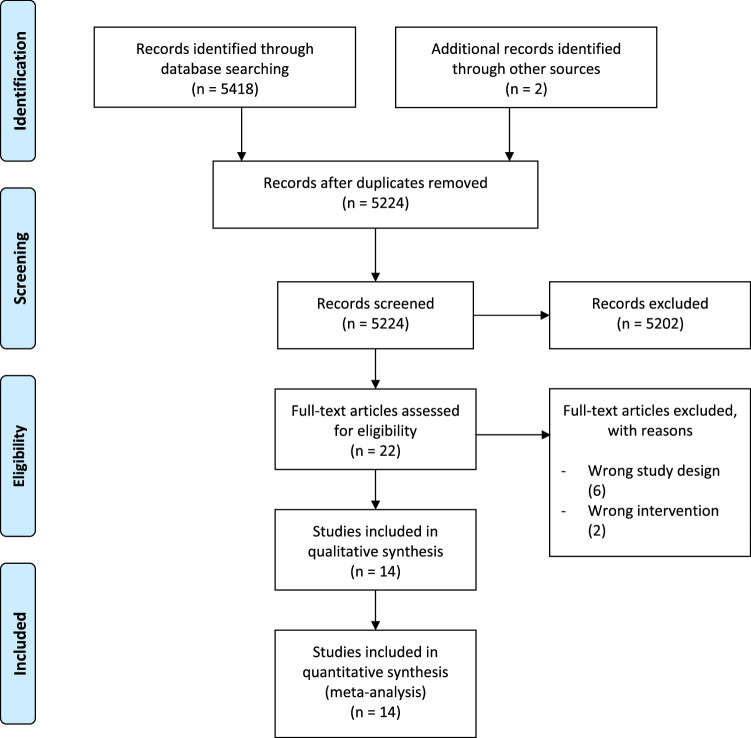
PRISMA flow diagram.

This systematic review and meta-analysis of 14 comparative studies involved a total of 12 625 patients. Out of 14 studies, 11 were randomized studies, and others were nonrandomized comparative studies. The search of databases yielded 5418 studies, and an additional 2 studies were added from other sources. After the removal of duplicates, 5224 studies were screened, and after the screening, 14 studies were identified as matches. Details are shown in Figure 1.

### Qualitative synthesis

This systematic review and meta-analysis included a total of 14 comparative studies that were published from 1982 to 2024. Out of 14 studies, 12 performed wound repair and suturing, while two performed urological procedures. Details of the included studies in the Population, Intervention, Comparator, and Outcome (PICO) format are shown in Table 1.

**Table 1:** Details of the included studies.

Study No.	Study details	Population (N)	Intervention (T)	Comparison (C)	Outcomes
1	Bodiwala *et al.*, 1982^5^ Design: Randomized clinical trial Procedure: Wound suturing Use of prophylactic antibiotics: Yes Country: United Kingdom	*N*=417 (T=210, C=207)	Performed without wearing sterile gloves (clean)	Performed by wearing sterile gloves (sterile)	Surgical site infection Overall infection: T=36/206 (17.47%), C=35/202 (17.32%) Mild infection: T=27/36 (75%), C=27/35 (77.14%) Severe infection: T=9/36 (25%), C=8/35 (22.85%)
2	Carapeti *et al.*, 1996^13^ Design: Randomized clinical trial Procedure: Urethral catheterization Use of prophylactic antibiotics: Unclear Country: United Kingdom	*N*=156 (T=82, C=74) Male=72/156 Female=84/156 Value (mean): Age (years): T=65.3, C=66.8	Performed without wearing sterile gloves (clean)	Performed by wearing sterile gloves (sterile)	Urinary tract infection Overall infection: T=9/82 (10.97%), C=7/74 (9.45%) Cost of catheterization (sterling pound): T=3.06, C=7.49 Cost of gloves (sterling pound): T=0.09, C=0.91
3	Fukushima *et al.*, 2024^14^ Design: Randomized clinical trial Procedure: Cystoscopy Use of prophylactic antibiotics: No Country: Japan	*N*=2634 (T=1376, C=1258) Male T=1130/1376, C=1045/1258 Female T=246/1376, C=213/20 Value [(median (IQR)] Age (years): T=72 (28-92), C=72 (28-92) Co-morbidities Diabetes mellitus: T=231/1376, C=224/1258 Cardiovascular disease: T=312/1376, C=301/1258 Neurological disease: T=79/1376, C=87/1258 Urinary findings Bloody urine: T=158/1376, C=157/1258 Purulent urine: T=207/1376, C=180/1258	Performed without wearing sterile gloves (clean)	Performed by wearing sterile gloves (sterile)	Urinary tract infection Overall infection: T=6/1376 (0.43%), C=6/1258 (0.47%)
4	Ghafouri *et al.*, 2014^15^ Design: Randomized clinical trial Procedure: Wound suturing Use of prophylactic antibiotics: Yes Country: Iran	*N*=186 (T=87, C=99) Male T=80/87, C=86/99 Female T=7/87, C=13/99 Value (mean±SD) Age (years): T=29.29±16, C=26.6±10 Wound size (cm): T=4.02±2.6, C=4.19±2.8 Wound location Head and neck: T=48/87, C=42/99 Limb: T=37/87, C=57/99 Trunk: T=2/87, C=0 Wound form Sharp: T=51/87, C=66/99 Blunt: T=36/87, C=33/99	Performed without wearing sterile gloves (clean)	Performed by wearing sterile gloves (sterile)	Surgical site infection Overall infection: T=4/87 (4.59%), C=2/99 (2.02%)
5	Heal *et al.*, 2015^16^ Design: Randomized clinical trial Procedure: Minor skin excision followed by wound suturing Use of prophylactic antibiotics: No Country: Australia	*N*=478 (T=241, C=237) Male T=142/241, C=143/237 Female T=99/241, C=94/237 Value (mean±SD) Age (years): T=64.9±15.8, C=65.7±15.3 Smoking status Never smoked: T=139/241, C=125/237 Ex-smoker: T=74/241, C=85/237 Current smoker: T=28/241, C=27/237 Medications Warfarin: T=10/241, C=12/237 Clopidogrel/aspirin: T=69/241, C=64/237 Steroids: T=15/241, C=19/237 Lesion characteristics Body site Neck and face: T=85/241, C=74/237 Upper extremities: T=65/241, C=72/237 Trunk: T=45/241, C=47/237 Lower limb above knee: T=11/241, C=4/237 Lower limb below knee: T=35/241, C=40/237 Histology Nevus or seborrheic keratosis: T=37/241, C=31/237 Skin cancer and precursor: T=160/241, C=167/237 Other: T=44/241, C=39/237	Performed without wearing sterile gloves (clean)	Performed by wearing sterile gloves (sterile)	Surgical site infection Overall infection: T=21/241 (8.71%), C=22/237 (9.28%)
6	Maitra *et al.*, 1986^6^ Design: Randomized clinical trial Procedure: Wound suturing Use of prophylactic antibiotics: Unclear Country: United Kingdom	*N*=230 (T=121, C=109) Male T=86/121, C=74/109 Female T=35/121, C=35/109 Number of study wounds: T=121, C=121 Value (mean±SD) Age (years): T=32±7.6, C=30±7.1	Performed without wearing sterile gloves (clean)	Performed by wearing sterile gloves (sterile)	Surgical site infection Overall infection: T=17/121 (14.04%), C=18/121 (14.87%) Grade I infection: T=9/17 (52.94%), C=12/18 (66.67%) Grade II infection: T=2/17 (11.76%), C=5/18 (27.78%) Grade III infection: T=6/17 (35.29%), C=1/18 (5.56%)
7	Mehta *et al.*, 2014^17^ Design: Nonrandomized clinical trial Procedure: Mohs surgery followed by wound suturing Use of prophylactic antibiotics: No Country: United States of America	*N*=1883 (T=941, C=942) Number of study wounds: T=1021, C=1004 Male T=577/1021, C=590/1004 Female T=444/1021, C=414/1004 Value (mean) Age (years): T=70.5, C=69.9 Preoperative defect size (cm^2^): T=2.71, C=2.59 Postoperative defect size (cm^2^): T=3.32, C=3.5 Lesion characteristics Body site Scalp: T=61/1021, C=72/1004 Forehead: T=166/1021, C=196/1004 Cheek or chin: T=203/1021, C=210/1004 Eye: T=50/1021, C=64/1004 Nose: T=240/1021, C=216/1004 Ear: T=95/1021, C=71/1004 Lip: T=41/1021, C=36/1004 Neck: T=38/1021, C=39/1004 Trunk: T=57/1021, C=44/1004 Extremities: T=46/1021, C=29/1004 Hands or feet: T=24/1021, C=26/1004 Pelvic area: T=0, C=1/1004 Histology Basal cell carcinoma: T=703/1021, C=676/1004 Squamous cell carcinoma: T=242/1021, C=180/1004 Squamous cell carcinoma in situ: T=61/1021, C=134/1004 Keratocarcinoma: T=4/1021, C=2/1004 Malignant melanoma in situ: T=4/1021, C=1/1004 Other: T=7/1021, C=11/1004	Performed without wearing sterile gloves (clean)	Performed by wearing sterile gloves (sterile)	Surgical site infection Overall infection: T=6/929 (0.64%), C=5/890 (0.56%) Cost of gloves (USD): T=13.60, C=37.30
8	Michener *et al.*, 2019^18^ Design: Randomized clinical trial Procedure: Excisions followed by wound suturing Use of prophylactic antibiotics: No Country: United States of America	*N*=93 (T=45, C=48) Male T=32/45, C=37/48 Female T=13/45, C=11/48 Value (mean±SD) Age (years): T=58.0±16.2, C=61.5±16.7 Total surgical time (min): T=39.7±16.7, C=39.6±15.3 Body site Head/Neck: T=16/45, C=15/48 Lower extremity: T=1/45, C=3/48 Trunk: T=24/45, C=23/48 Upper extremity: T=12/45, C=12/48	Performed without wearing sterile gloves (clean)	Performed by wearing sterile gloves (sterile)	Surgical site infection Overall infection: T=1/45 (2.22%), C=2/48 (4.16%)
9	Perelman *et al.*, 2004^19^ Design: Randomized clinical trial Procedure: Wound suturing Use of prophylactic antibiotics: No Country: Canada	N=816 (T=408, C=408) Male T=296/408, C=299/408 Female T=112/408, C=109/408 Value (mean±SD) Age (years): T=30.2±18.2, C=30.5±19.1 Time to repair (hour): T=2.57±1.62, C=2.55±1.39 Body site Head or Neck: T=149/408, C=150/408 Extremities: T=251/408, C=253/408 Trunk or buttocks: T=8/408, C=5/408	Performed without wearing sterile gloves (clean)	Performed by wearing sterile gloves (sterile)	Surgical site infection Overall infection: T=17/396 (4.29%), C=24/402 (5.97%)
10	Rinehart *et al.*, 2006^20^ Design: Retrospective study Procedure: Mohs surgery followed by wound suturing Use of prophylactic antibiotics: No Country: United States of America	*N*=1239 (T=679, C=560) Male T=500/679, C=381/560 Female T=179/679, C=179/560 Value (mean) Age (years): T=67.0, C=66.7 Tumor type Basal cell carcinoma: T=561/679, C=475/560 Squamous cell carcinoma: T=166/679, C=135/560 Melanoma: T=22/679, C=17/560 Others: T=17/679, C=7/560 Body site Eyelid: T=50/679, C=43/560 Lip: T=55/679, C=35/560 Ear: T=148/679, C=108/560 Nose: T=272/679, C=249/560 Cheek/chin: T=120/679, C=88/560 Forehead: T=83/679, C=178/560 Scalp: T=11/679, C=14/560 Neck: T=10/679, C=7/560 Extremity: T=14/679, C=10/560 Trunk: T=2/679, C=2/560 Genitalia: T=1/679, C=0	Performed without wearing sterile gloves (clean)	Performed by wearing sterile gloves (sterile)	Surgical site infection Overall infection: T=14/679 (2.06%), C=11/560 (1.96%) Cost per pair of gloves (USD): T=0.12, C=1.07
11	Wang *et al.*, 2023^21^ Design: Nonrandomized clinical trial Procedure: Oculoplastic surgery Use of prophylactic antibiotics: Yes Country: United States of America	N=3129 (T=1559, C=1570) Male T=655/1559, C=659/1570 Female T=904/1559, C=911/1570 Value (median) Age (years): T=58, C=60 Procedure Eyelid biopsy: T=588/1559, C=588/1570 Chalazion excision: T=384/1559, C=384/1570 Upper eyelid blepharoplasty: T=160/1559, C=161/1570 Müller’s muscle-conjunctival resection: T=133/1559, C=134/1570 Involutional entropion repair: T=87/1559, C=87/1570 Involutional ectropion repair: T=71/1559, C=72/1570 Subconjunctival orbital fat prolapse debulking: T=31/1559, C=31/1570 External levator advancement: T=20/1559, C=21/1570 Permanent tarsorrhaphy: T=12/1559, C=13/1570 Temporary tarsorrhaphy: T=11/1559, C=11/1570 Conjunctival biopsy: T=10/1559, C=11/1570 Lacrimal gland biopsy: T=10/1559, C=11/1570 Temporal artery biopsy: T=8/1559, C=9/1570 Canaliculotomy: T=8/1559, C=8/1570 Upper eyelid weight: T=8/1559, C=8/1570 Upper eyelid retraction repair: T=6/1559, C=7/1570 Second-stage Hughes flap: T=4/1559, C=5/1570 Tarsal fracture for cicatricial upper eyelid entropion: T=4/1559, C=5/1570 Dacryops marsupialization: T=3/1559, C=3/1570 Direct brow lift: T=1/1559, C=1/1570	Performed without wearing sterile gloves (clean)	Performed by wearing sterile gloves (sterile)	Surgical site infection Overall infection: T=1/1559 (0.06%), C=1/1570 (0.06%)
12	Worrall *et al.*, 1989^22^ Design: Randomized clinical trial Procedure: Wound suturing Use of prophylactic antibiotics: No Country: Canada	*N*=50 (T=25, C=25)	Performed without wearing sterile gloves (clean)	Performed by wearing sterile gloves (sterile)	Surgical site infection Overall infection: T=3/21 (14.28%), C=10/22 (45.45%)
13	Xia *et al.*, 2011^2^ Design: Randomized clinical trial Procedure: Mohs surgery followed by wound suturing Use of prophylactic antibiotics: Unclear Country: United States of America	*N*=60 (T=30, C=30) Male T=26/30, C=28/30 Female T=4/30, C=2/30 Value [median (IQR)] Age (years): T=71 (28–89), C=76 (47–88) Final defect size (cm^2^): T=2.0 (0.64–9.4), C=2.4 (0.60–16) Total surgical time (minutes): T=170 (120–240), C=180 (130–240) Body site Head and Neck: T=26/30, C=25/30 Nose: T=1/30, C=1/30 Ear: T=1/30, C=1/30 Trunk: T=2/30, C=2/30 Extremities: T=0, C=1/30	Performed without wearing sterile gloves (clean)	Performed by wearing sterile gloves (sterile)	Surgical site infection Overall infection: T=1/30 (3.33%), C=2/30 (6.67%)
14	Zwaans *et al.*, 2022^23^ Design: Randomized clinical trial Procedure: Wound suturing Use of prophylactic antibiotics: Unclear Country: Netherlands	*N*=1480 (T=733, C=747) Male T=535/733, C=554/747 Female T=198/733, C=193/747 Value (mean±SD) Age (years): T=39.5±16.9, C=39.2±16.5 Body site Head/Neck: T=275/733, C=253/747 Chest: T=0, C=6/747 Back: T=1/733, C=6/747 Nates: T=5/733, C=1/747 Arms: T=73/733, C=104/747 Hands: T=278/733, C=285/747 Legs: T=75/733, C=71/747 Feet: T=22/733, C=17/747 Unknown: T=4/733, C=4/747 Injury type Cut/sharp: T=411/733, C=425/747 Burst: T=311/733, C=310/747 Stab: T=4/733, C=7/747 Unknown: T=7/733, C=5/747 Wound size <2 cm: T=320/733, C=316/747 2-7 cm: T=382/733, C=400/747 >7 cm: T=29/733, C=27/747 Unknown: T=5/733, C=4/747 Contamination Not visible: T=616/733, C=634/747 Blood/clot: T=62/733, C=68/747 Asphalt: T=12/733, C=7/747 Glass: T=0, C=1/747 Dirt: T=7/733, C=2/747 Other: T=25/733, C=15/747 Unknown: T=11/733, C=20/747	Performed without wearing sterile gloves (clean)	Performed by wearing sterile gloves (sterile)	Surgical site infection Overall infection: T=38/667 (5.69%), C=46/673 (6.83%)

### Quantitative synthesis

#### Overall postprocedural infection

**Figure 2:**
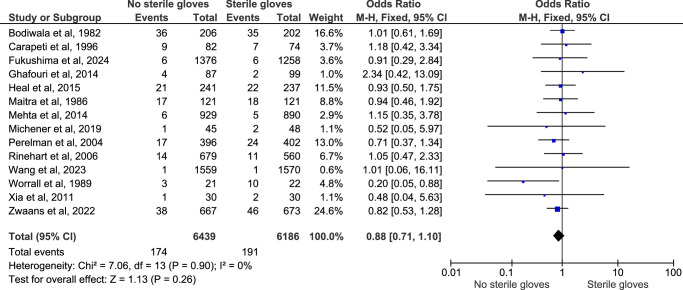
Forest plot for overall postprocedural infection outcome.

**Figure 3:**
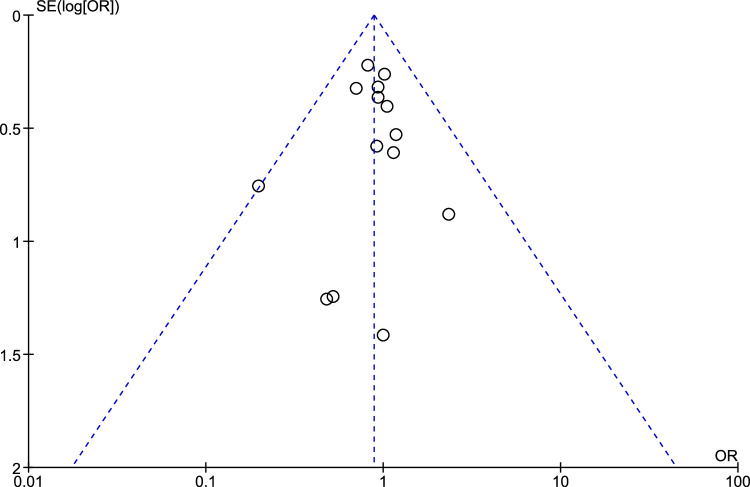
Funnel plot for overall postprocedural infection. outcome.

Postprocedural infection was reported in all 14 studies, and pooling of the data using the fixed effect model showed that there was no significant difference between the two groups (OR: 0.88; 95% CI: 0.71–1.10; *n*=12 625; *I* ^2^=0%; *P*-value=0.26) (Fig. 2). The sensitivity analysis of this outcome did not show any significant difference in the result. Also, the funnel plot that was used to assess the publication bias showed a symmetrical plot, denoting no publication bias for this outcome (Fig. 3). This showed that there is no difference between the postprocedural outcome of performing minor surgical procedures without using sterile gloves (clean method) and by using sterile gloves (sterile method).

#### Postprocedural infection among randomized studies

**Figure 4:**
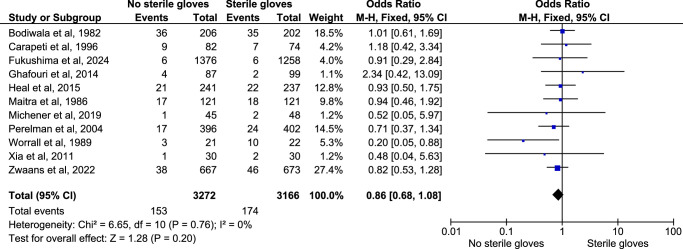
Funnel plot for subgroup analysis of randomized. studies only.

The subgroup analysis of the postprocedural infection reported among 11 randomized clinical trials showed no significant difference between the two groups (OR: 0.86; 95% CI: 0.68–1.08; *n*=6438; *I* ^2^=0%; *P*-value=0.20) (Fig. 4). The sensitivity analysis of this outcome did not show any significant difference in the result, and the funnel plot showed a symmetrical plot denoting no publication bias for this outcome (Figure C, Supplementary File 2, Supplemental Digital Content 5, http://links.lww.com/JS9/D178). This showed that performing minor surgical procedures without using sterile gloves (clean method) and by using sterile gloves (sterile method) had no difference in the postprocedural infection outcome.

#### Postprocedural infection with no prophylactic antibiotics

**Figure 5:**
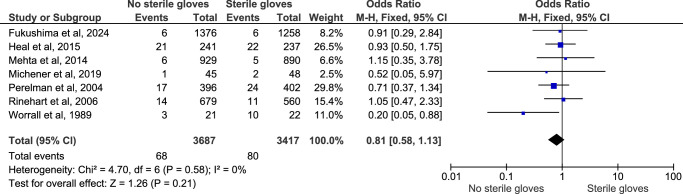
Funnel plot for subgroup analysis of patients that did. not receive prophylactic antibiotics.

The subgroup analysis of the postprocedural infection outcome among patients who did not receive prophylactic antibiotics showed no significant difference between the two groups (OR: 0.81; 95% CI: 0.58–1.13; *n*=7104; *I* ^2^=0%; *P*-value=0.21) (Fig. 5). The sensitivity analysis of this outcome did not show any variation.

#### Surgical site infection

**Figure 6:**
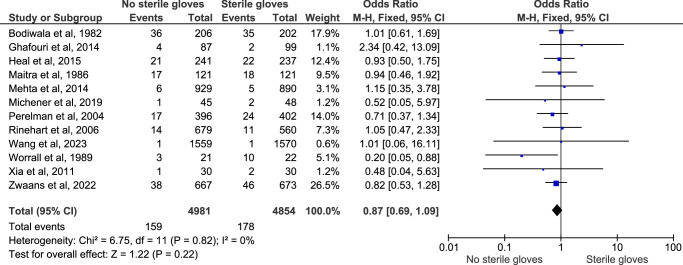
Funnel plot for subgroup analysis of surgical site. infection.

The subgroup analysis of the surgical site infection reported among 12 comparative studies showed no significant difference in the infection rate between the two groups (OR: 0.87; 95% CI: 0.69–1.09; *n*=9835; *I* ^2^=0%; *P*-value=0.22) (Fig. 6). The sensitivity analysis of this outcome did not show any significant difference and the funnel plot yielded symmetrical plot (Figure D, Supplementary File 2, Supplemental Digital Content 5, http://links.lww.com/JS9/D178).

#### Infection after urological infection

**Figure 7:**
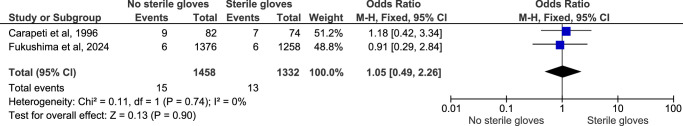
Funnel plot for subgroup analysis of urological. procedure.

Infection after urological procedure was reported among two comparative studies and this subgroup analysis showed no significant difference in the infection rate between the two groups (OR: 1.05; 95% CI: 0.45–2.26; *n*=2790; *I* ^2^=0%; *P*-value=0.90) (Fig. 7).

## Discussion

This systematic review and meta-analysis of 14 studies included studies that compared the outcomes of using sterile gloves to those of without using sterile gloves in various minor surgical procedures like excision and suturing, wound repair, urinary catheterization, and cystoscopy. In this study, sensitivity analysis and subgroup analysis were also performed to assess the robustness of the results.

This study assessed the overall postprocedural infection outcome, and it was found that there was no difference in infection rate when the procedure was performed with sterile gloves or without sterile gloves. There was no heterogeneity in this analysis; the sensitivity analysis showed no difference in the result; and the funnel plot also indicated that there was no publication bias associated with the analysis. This inferred that, with certainty, the use of sterile gloves while performing minor surgical procedures has no additional benefit regarding the postprocedural infection outcome. The majority of the included studies were from developed nations (13 out of 14), while only one study was from a lower-middle-income country. This did not make the analysis of the variation of the outcomes according to the economic state of the country feasible. Subgroup analysis with only randomized controlled trials included also showed no difference in postprocedural infection with no heterogeneity, no publication bias, and no variation in sensitivity analysis. This result is in unison with the findings of Tan *et al*.^24^, where they studied wound repair in an emergency department and similar findings have been reported in the findings of various studies from the late 1980s to 2024^6,14,16,22^. Also, the subgroup analysis of wound repair and urological procedures showed no difference in the infection rate. Not only while performing minor surgical procedures, randomized controlled trial that compared the outcomes of using sterile gloves and nonsterile gloves while performing dental procedures and obstetrical procedures also showed no difference^25,26^.

Morshedi *et al*., in their study, concluded that using nonsterile gloves was unlikely to increase the risk of infection, and their use could cut the cost by up to 92%, saving up to US $25 000 per year at their centre^26^. A quantitative comparison of the cost of care could not be made due to the unavailability of data, but among the included studies, two reported the cost of gloves, and both studies reported that the cost of sterile gloves can be many folds higher than that of nonsterile gloves^17,20^. The cost of gloves can differ from centre to centre, but in comparison, a pair of sterile gloves costs more than a pair of nonsterile gloves, which can increase the total cost of care. This is felt even more in low-to-middle-income countries (LMIC), where patients have to bear all the costs at the local level and the country has to spend its reserve to import more expensive sterile gloves. Cost saving by diligent use of gloves may seem small per patient but cumulative effort for many years become significant as minor surgical procedures are commonly performed^24^. At the time of the crisis, like during the COVID-19 pandemic, many centers faced a lack of medical equipment, including gloves^27^ and this goes to show that even if it can be afforded, the diligent and evidence-based use of resource is the only justification.

The flip side of the overuse and unnecessary use of medical resources is that it poses a risk of environmental hazards. Hospital waste is one of the major contributing factors to environmental pollution, but hospitals also contribute to carbon emissions in a major way^28^. Jamal *et al*.^29^ studied the impact of sterile gloves over the environment and found that sterile latex gloves had 11.6 times higher impact over the environment and sterile gloves had greater contribution in climate change, ozone depletion, and ionizing radiation emission. By recognizing such risks of hospital waste and rampant use of resources, The Royal College of Surgeons of United Kingdom have released the Intercollegiate Green Theater Checklist, which promotes and advocates for diligent and sustainable use of resources^7^.

This systematic review and meta-analysis showed that the use of sterile gloves does not have an extra benefit, and there is no difference in postprocedural infection outcome between the groups using sterile gloves and not using sterile gloves while performing minor surgical procedures. Minor surgical procedures are limited to excision and suturing, wound repair, urinary catheterization, and cystoscopy. This finding should not be extrapolated to perform more complex and invasive procedures. Also, it needs to be acknowledged that the included studies had patients who were immunocompetent.

### Limitations

The included studies studied the outcome of different minor surgical procedures, and there was also no uniformity in the prophylactic antibiotic use among the included studies. This was the limitation of this study, and to address this issue, a subgroup analysis was performed. Similarly, there was variation in the anatomical location in which the procedure was performed. Regarding the wound closure procedure, the suture material and suture placed were different. Also, only one of the included studies mentioned diabetes as the co-morbidity. Further studies that can compare the outcomes performed for areas that are more prone to infections, like intertriginous sites, are needed to further consolidate the findings of this systematic review and meta-analysis.

## Conclusion

There is no difference in the postprocedural infection outcome when minor surgical procedures are performed using the sterile glove and without using the sterile gloves. Use of sterile gloves do not have extra benefit for preventing infections when minor surgical procedures are performed.

## Ethical approval

Ethical approval was not required for this systematic review and meta-analysis.

## Consent

Informed consent was not required for this systematic review and meta-analysis.

## Source of funding

Not applicable.

## Author contribution

O.S. and S.B.: contributed to the conceptualization of the study; N.B., S.B., and N.T.: contributed to the screening of the studies; N.B., N.T., N.J., and S.P.: were involved in the data curation phase; O.S. and S.B.: did the formal analysis; O.S., A.B., B.M., A.A., and N.S.: were involved in initial manuscript drafting; S.B.: edited the manuscript intellectually. All the authors were involved in approving the final version of the manuscript.

## Conflicts of interest disclosure

The authors declares no conflicts of interest.

## Research registration unique identifying number (UIN)

Protocol followed in this study was prospectively registered in the Registry of Systematic Reviews/Meta-Analyses (Unique Identifying Number: reviewregistry1818) URL: https://www.researchregistry.com/register-now/register-your-systematicreview#registryofsystematicreviewsmeta-analyses/registryofsystematicreviewsmetaanalysesdetails/6612a85bd961d500273d5e0a/ Protocol followed in this study was prospectively registered in the International prospective register of systematic reviews (PROSPERO ID: CRD42024505779). URL: https://www.crd.york.ac.uk/prospero/display_record.php?RecordID=505779.

## Guarantor

Oshan Shrestha, MBBS Nepalese Army Institute of Health Sciences, Kathmandu, Nepal. E-mail: shresthaoshan93@gmail.com.

## Data availability statement

The analyzed data is available in the manuscript. All the data are presented in Table 1.

## Provenance and peer review

Not commissioned, externally peer-reviewed.

## Supplementary Material
