# Supplementary material for: Postprocedural infection rate after minor surgical procedures performed with and without sterile gloves: a systematic review and meta-analysis
Source: Int J Surg. 2024 Jul 24;110(11):7341–52. doi: 10.1097/JS9.0000000000001993 (PMC11573057; doi:10.1097/JS9.0000000000001993)
Supplement: Supplementary file 2 [file js9-110-7341-s002.docx]

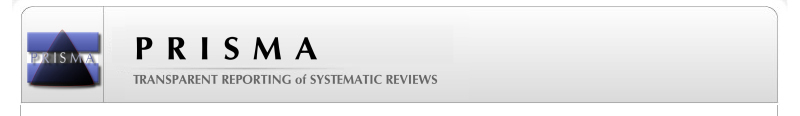
**PRISMA 2009 Flow Diagram**

Studies included in quantitative synthesis (meta-analysis)
(n = 14)

Studies included in qualitative synthesis
(n = 14)

Full-text articles assessed for eligibility
(n = 22)

Records excluded
(n = 5202)

Records screened
(n = 5224)

Records after duplicates removed
(n = 5224)

Additional records identified through other sources
(n = 2)

## Identification

## Eligibility

## Included

## Screening

Records identified through database searching
(n = 5418)

Full-text articles excluded, with reasons

- Wrong study design (6)
- Wrong intervention (2)
