# Supplementary material for: Postprocedural infection rate after minor surgical procedures performed with and without sterile gloves: a systematic review and meta-analysis
Source: Int J Surg. 2024 Jul 24;110(11):7341–52. doi: 10.1097/JS9.0000000000001993 (PMC11573057; doi:10.1097/JS9.0000000000001993)
Supplement: Supplementary file 4 [file js9-110-7341-s004.docx]

**Search details**

Search date: January 24^th^, 2024

PubMed:

(sterile) AND ("clean" OR "unsterile" OR "nonsterile") AND (gloves) AND ("dressing" OR "procedure" OR "outpatient" OR "surgery")

Hits: 103

Link: <https://pubmed.ncbi.nlm.nih.gov/?term=%28sterile%29+AND+%28%22clean%22+OR+%22unsterile%22+OR+%22nonsterile%22%29+AND+%28gloves%29+AND+%28%22dressing%22+OR+%22procedure%22+OR+%22outpatient%22+OR+%22surgery%22%29>+

PubMed Central

"sterile"[All Fields] AND ("clean"[All Fields] OR "unsterile"[All Fields] OR "nonsterile"[All Fields]) AND ("gloves, protective"[MeSH Terms] OR ("gloves"[All Fields] AND "protective"[All Fields]) OR "protective gloves"[All Fields] OR "gloves"[All Fields]) AND ("dressing"[All Fields] OR "procedure"[All Fields] OR "outpatient"[All Fields] OR "surgery"[All Fields])

Hits: 4950

Link: <https://www.ncbi.nlm.nih.gov/pmc?term=%22sterile%22%5BAll%20Fields%5D%20AND%20%28%22clean%22%5BAll%20Fields%5D%20OR%20%22unsterile%22%5BAll%20Fields%5D%20OR%20%22nonsterile%22%5BAll%20Fields%5D%29%20AND%20%28%22gloves%2C%20protective%22%5BMeSH%20Terms%5D%20OR%20%28%22gloves%22%5BAll%20Fields%5D%20AND%20%22protective%22%5BAll%20Fields%5D%29%20OR%20%22protective%20gloves%22%5BAll%20Fields%5D%20OR%20%22gloves%22%5BAll%20Fields%5D%29%20AND%20%28%22dressing%22%5BAll%20Fields%5D%20OR%20%22procedure%22%5BAll%20Fields%5D%20OR%20%22outpatient%22%5BAll%20Fields%5D%20OR%20%22surgery%22%5BAll%20Fields%5D%29&cmd=DetailsSearch>

EMBASE:

sterile AND ('clean' OR 'unsterile' OR 'nonsterile') AND ('gloves'/exp OR gloves) AND ('dressing'/exp OR 'dressing' OR 'procedure'/exp OR 'procedure' OR 'outpatient'/exp OR 'outpatient' OR 'surgery'/exp OR 'surgery')

Hits: 275

Link: <https://www.embase.com/#advancedSearch/resultspage/history.3/page.1/25.items/orderby.date/source>.

SCOPUS:

( sterile ) AND ( "clean" OR "unsterile" OR "nonsterile" ) AND ( gloves ) AND ( "dressing" OR "procedure" OR "outpatient" OR "surgery" )

Hits: 90

Link: <https://www.scopus.com/results/results.uri?sort=plf-f&src=s&st1=%28sterile%29+AND+%28%22clean%22+OR+%22unsterile%22+OR+%22nonsterile%22%29+AND+%28gloves%29+AND+%28%22dressing%22+OR+%22procedure%22+OR+%22outpatient%22+OR+%22surgery%22%29&sid=b27eb1500ed4ab49d182462880363035&sot=b&sdt=b&sl=143&s=TITLE-ABS-KEY%28%28sterile%29+AND+%28%22clean%22+OR+%22unsterile%22+OR+%22nonsterile%22%29+AND+%28gloves%29+AND+%28%22dressing%22+OR+%22procedure%22+OR+%22outpatient%22+OR+%22surgery%22%29%29&origin=searchbasic&editSaveSearch=&yearFrom=Before+1960&yearTo=Present&sessionSearchId=b27eb1500ed4ab49d182462880363035&limit=10>
