# Supplementary material for: Postprocedural infection rate after minor surgical procedures performed with and without sterile gloves: a systematic review and meta-analysis
Source: Int J Surg. 2024 Jul 24;110(11):7341–52. doi: 10.1097/JS9.0000000000001993 (PMC11573057; doi:10.1097/JS9.0000000000001993)

Figure A. Risk of bias summary of randomized studies

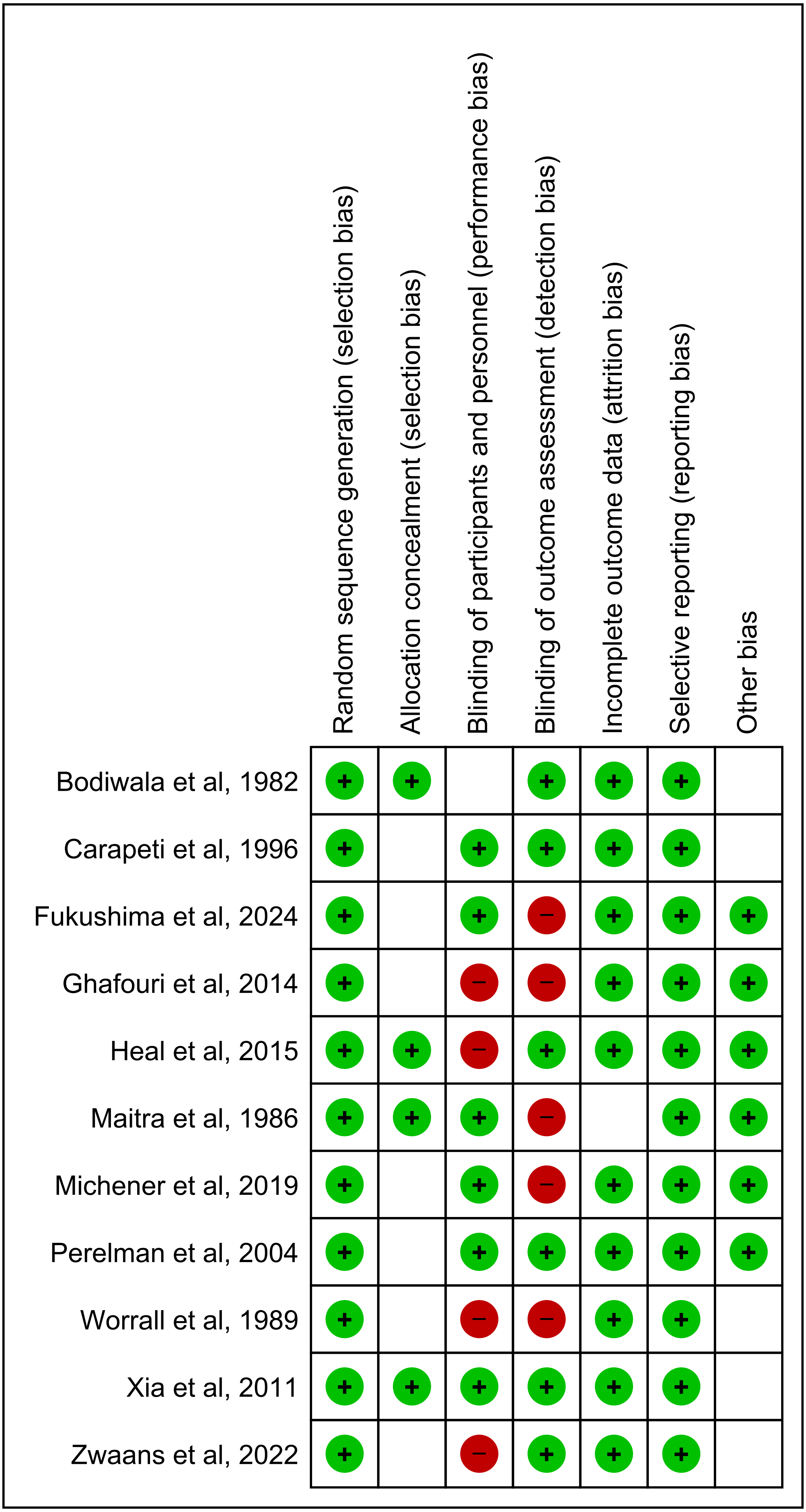

Figure B. ROBINS-I summary of non-randomized studies

Study	Bias due to confounding	Bias in selection of participants into the study	Bias in classification of interventions	Bias due to deviations from intended interventions	Bias due to missing data	Bias in measurement of outcomes	Bias in selection of the reported result	Overall risk of bias
Mehta et al, 2014	Moderate	Low	Low	Low	Low	Low	Low	Low
Rinehart et al, 2006	Moderate	Moderate	Low	Low	Low	Low	Low	Moderate
Wang et al, 2023	Moderate	Low	Low	Low	Moderate	Low	Low	Moderate

Figure C. Funnel plot for sub-group analysis of randomized studies only

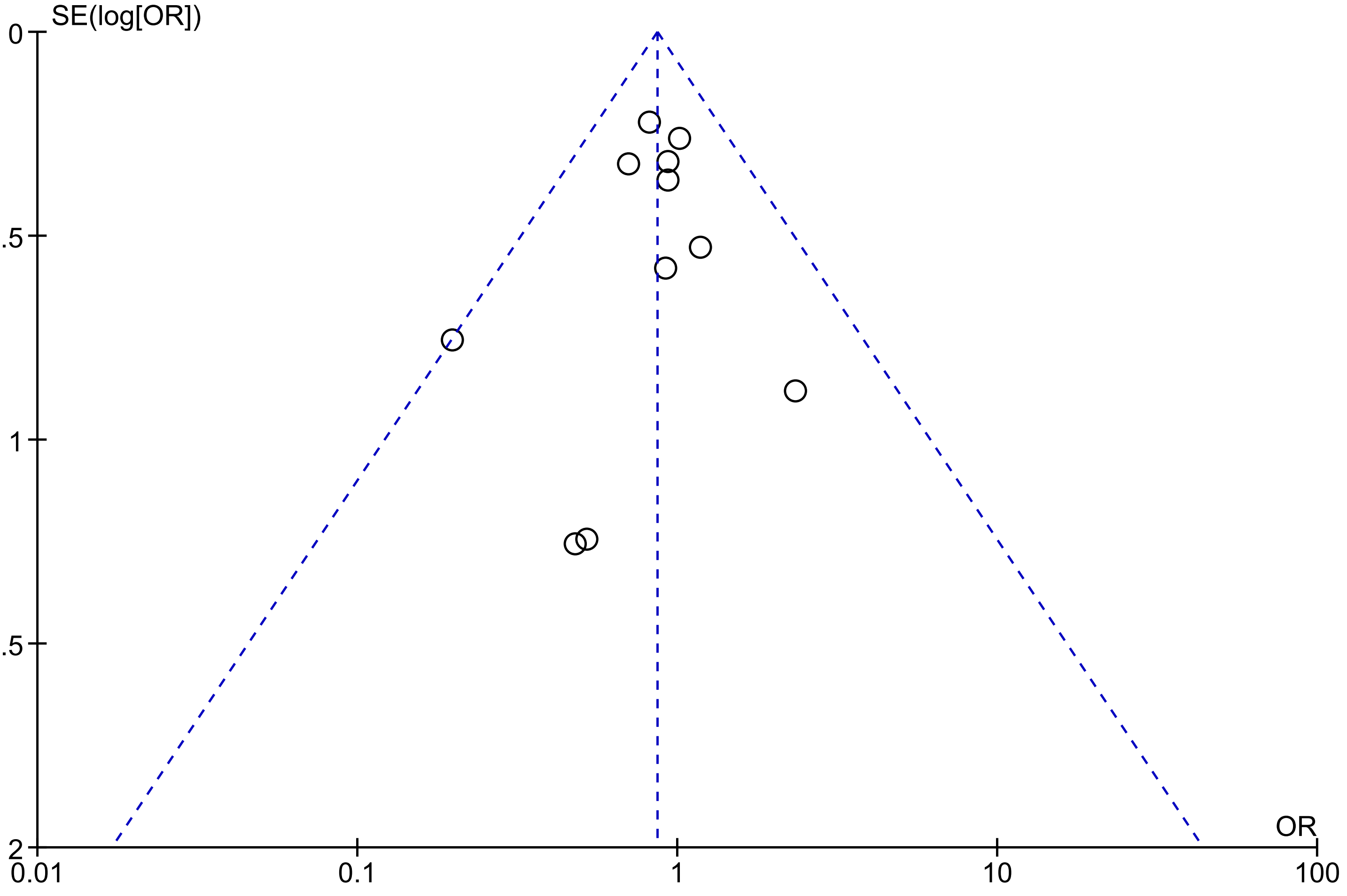

Figure D. Funnel plot for sub-group analysis of surgical site infection

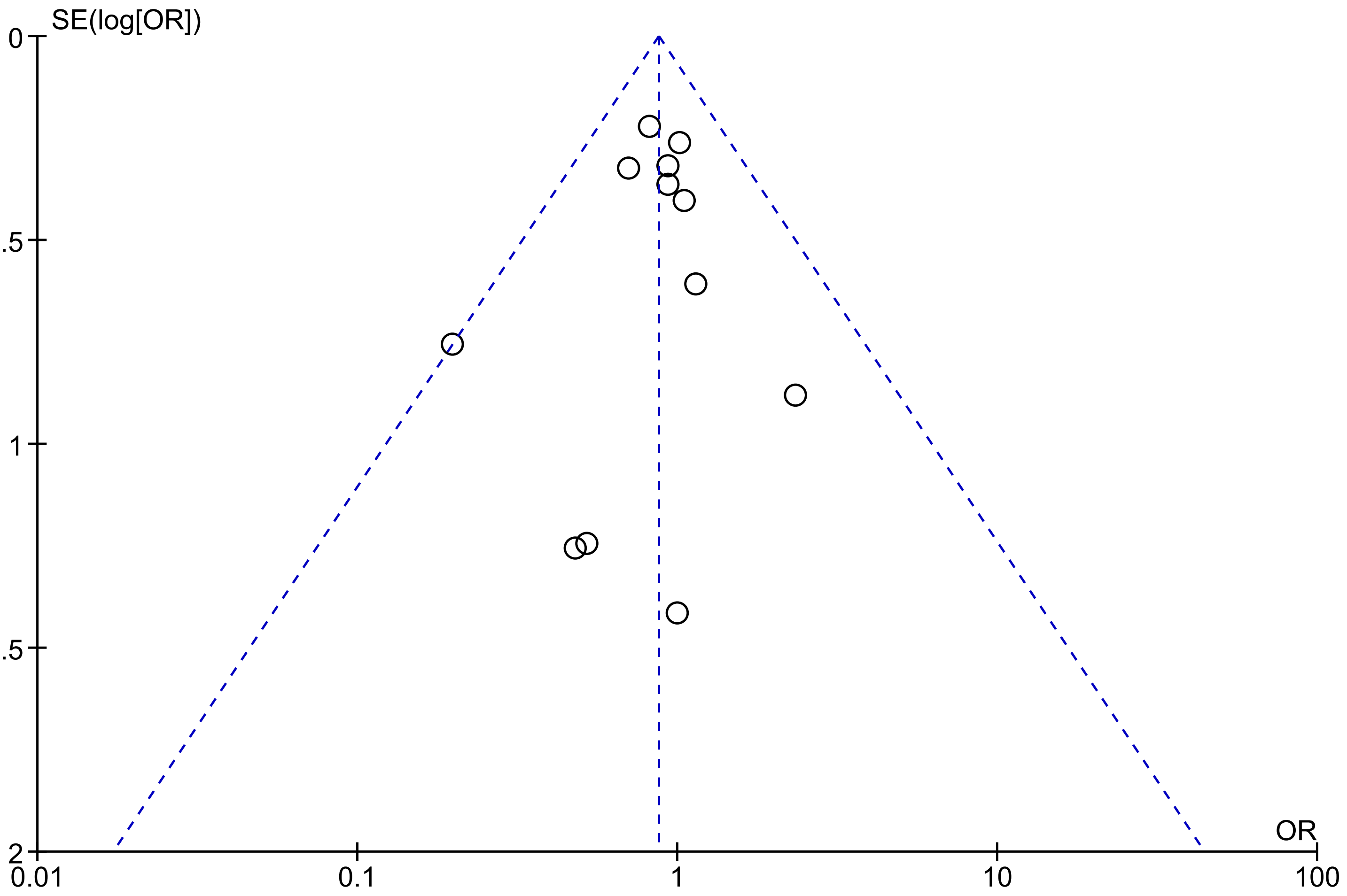

Supplement: Supplementary file 5 [file js9-110-7341-s005.pdf]
